# *Candida glabrata*: A Lot More Than Meets the Eye

**DOI:** 10.3390/microorganisms7020039

**Published:** 2019-01-30

**Authors:** Kundan Kumar, Fizza Askari, Mahima Sagar Sahu, Rupinder Kaur

**Affiliations:** 1 Laboratory of Fungal Pathogenesis, Centre for DNA Fingerprinting and Diagnostics, Hyderabad 500039, India; kundankumar@cdfd.org.in (K.K.); fizzaaskari@cdfd.org.in (F.A.); mahimasagar@cdfd.org.in (M.S.S.); 2 Graduate Studies, Manipal Academy of Higher Education, Manipal 576104, India; 3 Graduate Studies, Regional Centre for Biotechnology, Faridabad 121001, India

**Keywords:** *Candida glabrata*, yeast pathogens, adherence, biofilm formation, aspartyl proteases, stress response mechanisms, host immune cells

## Abstract

*Candida glabrata* is an opportunistic human fungal pathogen that causes superficial mucosal and life-threatening bloodstream infections in individuals with a compromised immune system. Evolutionarily, it is closer to the non-pathogenic yeast *Saccharomyces cerevisiae* than to the most prevalent *Candida* bloodstream pathogen, *C. albicans*. *C. glabrata* is a haploid budding yeast that predominantly reproduces clonally. In this review, we summarize interactions of *C. glabrata* with the host immune, epithelial and endothelial cells, and the ingenious strategies it deploys to acquire iron and phosphate from the external environment. We outline various attributes including cell surface-associated adhesins and aspartyl proteases, biofilm formation and stress response mechanisms, that contribute to the virulence of *C. glabrata*. We further discuss how, *C. glabrata*, despite lacking morphological switching and secreted proteolytic activity, is able to disarm macrophage, dampen the host inflammatory immune response and replicate intracellularly.

## 1. Introduction

*Candida glabrata* is an opportunistic human fungal pathogen that accounts for up to 29% of total *Candida* bloodstream infections [1,2]. Its prevalence varies with the geographic area [2,3,4]. While *C. glabrata* is the second most common bloodstream *Candida* species after *C. albicans* in Northern Europe and the USA [1,5,6], it ranks as the third or fourth most prevalent invasive *Candida* pathogen in Asia [4,6,7]. Recent epidemiological surveys have shown a decrease in the frequency of *C. albicans* and an increased prevalence of non-*albicans Candida* [3,5,6]. *C. glabrata* bloodstream infections are commonly found in elderly individuals, diabetic patients and solid organ transplant recipients [6,8,9]. *C. glabrata* is also a causative agent of urinary tract and vaginal infections [10,11].

Historically, *C. glabrata* was named *Cryptococcus glabrata*, and this name was first changed to *Torulopsis glabrata* and later to *Candida glabrata*. Since pseudohyphae formation was not found to be a dependable criterion to classify yeasts at the genus level, the *Torulopsis* and *Candida* genera were merged under the *Candida* genus name [10,12]. However, based on molecular evolutionary studies, *C. glabrata* was later assigned to the genus *Nakaseomyces* [13]. The current taxonomy of *C. glabrata* is the Kingdom *Fungi*, Subkingdom *Dikarya*, Phlyum *Ascomycota*, Subphylum *Saccharomycotina*, Class *Saccharomycetes*, Order *Saccharomycetales*, Family *Saccharomycetaceae*, Genus *Nakaseomyces*, Clade *Nakaseomyces/Candida* and Species *glabrata* (NCBI:txid284593).

Clinically, *C. glabrata* is primarily diagnosed via culture-based assays viz., colony color (white/pink/purple) on CHROMagar Candida medium and microscopic examination [presence of small-sized (1–4 μm) yeast cells and lack of hyphal structures], and biochemical methods, viz., assimilation of glucose and trehalose sugars [14]. Additionally, the FDA (Food and Drug Administration, USA)-approved T2 Candida panel, which can identify five *Candida* species, *C. albicans*, *C. glabrata*, *C. tropicalis*, *C. parapsilosis* and *C. krusei*, is increasingly being used for rapid *Candida* species detection in hospital settings [15,16]. In the current review, we summarize the genomic configuration, virulence traits and nutrient acquisition and stress signaling pathways in *C. glabrata*. Additionally, we provide an overview of *C. glabrata*-host cell interaction mechanisms.

## 2. Genomic Architecture

*C. glabrata* is a haploid budding yeast, and belongs to the non-CTG, Saccharomycetaceae clade in which the CTG codon encodes leucine [17,18]. Contrarily, *C. albicans* belongs to the CTG clade wherein the CTG codon is translated as serine instead of leucine [17,18]. The genome of the *C. glabrata* CBS138 strain was sequenced by the Genolevures Consortium using whole genome shotgun sequencing and represented by 1000 contigs [17]. The annotated genome contains 13 chromosomes, named as Chromosome A to M, and is of 12.3 Mb in size [17]. Of a total of 5293 open reading frames (ORFs) in the *C. glabrata* genome, only 238 (4.5%) ORFs are verified with some experimental evidence for the existence of their gene products [www.candidagenome.org]. The chromosome length varies from 4,91,328 to 14,55,689 bp with the chromosome A and L being the smallest and largest, respectively [www.candidagenome.org]. The *C. glabrata* genome shows plasticity, and alterations in karyotype and chromosome size have been observed in clinical isolates and the reference strain CBS138 [19,20,21]. 

The *C. glabrata* genome displays robust synteny with the genome of the non-pathogenic yeast *Saccharomyces cerevisiae*, and synteny blocks were found to be present in 88% of the genome of these two yeasts [17,22]. Consistently, *C. glabrata* contains orthologs of 4870 *S. cerevisiae* genes [https://yeastmine.yeastgenome.org/], however, it also possesses a set of 337 genes that are absent in *S. cerevisiae* [23]. Both *C. glabrata* and *S. cerevisiae* belong to the Whole Genome Duplication (WGD) group, and are assumed to have arisen from the same tetraploid hybrid ancestor, which existed about 100-200 million years ago [18]. This ancestor is thought to have undergone extensive whole genome duplication brought about by an interspecies hybridization event, which helped the hybrid to regain fertility [24]. Furthermore, compared to the *S. cerevisiae* lineage, a greater degree of gene loss has occurred in the *C. glabrata* lineage [17,18]. The *C. glabrata* genome consistently exhibits lower global redundancy [17]. 

Although the reductive genome evolution was thought to be linked with the pathogenic life style of *C. glabrata* with more reliance on the human host for nutrients [17], recent studies do not support this notion [18,25]. As mentioned earlier, within the Saccharomycetaceae family, *C. glabrata* belongs to the genus *Nakaseomyces*, which contains three pathogenic species (*C. glabrata*, *C. nivariensis* and *C. bracarenses*) and three environmental species (*C. castellii*, *Kluyveromyces delphensis* and *K. bacillisporus*) [18]. Of these, *C. castellii* and *K. bacillisporus* belong to one group, while the remaining four are represented by a group, referred to as the ‘glabrata group’ [18]. Intriguingly, molecular phylogenetic and genome analysis have revealed three human pathogenic species, *C. glabrata*, *C. nivariensis* and *C. bracarenses* of the glabrata group, to be polyphyletic in origin [18,25]. 

*C. glabrata* contains a small 20 kb circular mitochondrial genome which contains eleven ORFs, including genes coding for three subunits of the cytochrome C oxidase (CgCox1, 2 and 3), the apocytochrome b (CgCob) and three subunits of the ATP synthase (CgAtp6, 8 and 9) [26], (www.candidagenome.org). It also contains 23 tRNAs, 2 rRNAs and 1 non-coding RNA (www.candidagenome.org).

The reproduction mode in *C. glabrata* is predominantly clonal, and the opportunistic pathogenic lifestyle of *C. glabrata* is also thought to have arisen independently from that of *C. albicans* [25]. As discussed above, *C. glabrata* belongs to the post-WGD non-CTG clade and appears to be evolutionarily uniquely placed. Phylogenetically, it is closer to *S. cerevisiae* and only distantly related to the most common pathogenic fungus, *C. albicans* [17,18]. Furthermore, *Nakaseomyces* is the only group that possesses the ability to infect humans among the post-WGD clade, and, recently identified *C. bracarensis* and *C. nivariensis* are the pathogenic close relatives of *C. glabrata* [18]. As the capability to infect humans appears to have emerged independently within the *Nakaseomyces* [18], *C. glabrata* may have acquired a unique set of pathogenesis attributes. In this review, we discuss the unique features of *C. glabrata*, as well as the characteristics that it shares with the non-pathogenic yeast *S. cerevisiae* and the pathogenic yeast *C. albicans*. Of note, virulence mechanisms of *C. bracarensis* and *C. nivariensis* are yet to be identified. Table 1 summarizes the key characteristic features of *C. glabrata* and *C. albicans*. 

## 3. Salient Pathobiological Features

The traits that may contribute to the virulence of *C. glabrata*, are discussed below.

### 3.1. Adherence

Adherence to the host tissue is an important trait that contributes to colonization and establishment of successful infections. *C. glabrata* possesses a total of 67 putative adhesins, which may mediate adherence to host cells [27]. These putative glycosylphosphatidylinositol (GPI)-anchored cell wall proteins are composed of a N-terminal ligand-binding domain and a low complexity serine/threonine-rich region with internal tandem repeats followed by a C-terminus GPI-anchor attachment site [27]. Based on their putative ligand-binding regions, the adhesin gene family has been classified into seven sub-families [27]. The Epa (Epithelial Adhesin) sub-family I, containing the PA14 (Anthrax Protective Antigen) ligand-binding domain, is most well studied, and consists of 17 to 23 proteins depending upon the isolate [27,28]. The sequenced fecal isolate CBS 138 contains 17 Epa-encoding genes, while the widely used vaginal isolate BG2 contains 23 Epa-encoding genes [27,28]. The majority of Epa adhesins are encoded by sub-telomeric localized genes and regulated by epigenetic SIR (Silent Information Regulator)-dependent transcriptional silencing [27,29,30,31]. 

The founding member of the Epa adhesin family, Epa1, is a calcium-dependent lectin, and aids adhesion to epithelial cells [32] and macrophages [33]. The multidrug resistance transcription factor CgPdr1 has been implicated in regulation of the *EPA1* gene expression [34]. The Epa6 and Epa7 adhesins have been shown to mediate adherence to epithelial and endothelial cells [30,35]. Furthermore, *EPA6* was found to be expressed in the murine urinary tract infection model, due to unavailability of the nicotinic acid, which is a precursor for the CgSir2 histone deacetylase cofactor, nicotinamide adenine dinucleotide (NAD^+^) [30]. Similarly, differential expression of *EPA2*, *EPA3*, *EPA7* and *EPA22* genes has been reported in response to different environmental cues [36,37,38,39,40,41,42]. Recent analysis has linked expansion of the *EPA* gene family with virulence of the fungal species of the Nakaseomyces clade, with *C. glabrata*, *C. bracarensis*, *C. nivariensis* and *K. delphensis* containing 17–23, 12, 9 and 1 Epa adhesins, respectively [18].

With regard to ligands, Epa1, Epa6 and Epa7 were found to bind to oligosaccharides containing a terminal galactose residue [35]. Despite the preference for the terminal galactose, Epa1, 6 and 7 showed glycan ligand specificity, with Epa6 having the widest substrate specificity [35,43]. In accordance, a recent study has predicted many human receptors including mucins, CD43 (leukosialin)/CD45 (receptor-type tyrosine-protein phosphatase) glycoproteins, ceruloplasmin, (sero)transferrin, and fibronectin, for Epa1, 6 and 7 [44]. Furthermore, adherence analysis of *S. cerevisiae* strains expressing ligand-binding domains of 15 Epa adhesins individually revealed significant, moderate and very weak adherence to human epithelial colorectal adenocarcinoma cells (Caco2) for Epa1, 6 and 7, Epa8, 9, 12, 15 and 23, and Epa2, 3, 11, 13, 19, 20 and 21, respectively [45].

The second adhesin sub-family contains a N-terminal PA14 domain, referred to as the Pwp family (PA14-containing Wall Protein), and contains seven proteins. Of these Pwp1-7, the Pwp7 protein is required for adherence to human endothelial cells in vitro [23]. The number of adhesins in sub-families III, IV, V, VI and VII vary from 3 to 13, and are yet to be characterized for their ligands and functions [27]. Two adhesins, CAGL0L09911p and CAGL0J05159p/CAGL0J05170p, did not belong to any of the subfamily, and were grouped separately [27]. Many adhesins including Epa proteins contained 46-amino acids repeats (Awp2 repeats) in their C-terminus low-complexity regions [27]. One such protein, Aed1 (Adherence to Endothelial cells), belonging to the sub-family III, has been implicated in adherence to human endothelial cells [23]. Of note, a recent study has identified 49 novel protein-coding genes, of which eight are located in close proximity to *EPA* or *PWP* genes in the subtelomeric regions [46].

Importantly, many adhesin-encoding genes contain several kilobases of 126–429 bp-long sequences, that are tandemly repeated up to 32-times, called megasatellites [47]. The number of repeats in these mega-satellites may govern the length and functions of adhesions [47]. Moreover, *C. glabrata* clinical isolates are known to have varied number of adhesin-encoding genes, and a distinct profile of cell wall proteins expressed at the cell surface [41,48,49]. Therefore, the presence of such a large number of adhesin genes and their complex environment-dependent differential regulation are likely to help *C. glabrata* colonize different host niches as well as to form biofilms on a wide range of surfaces [27,47,48].

### 3.2. Biofilm Formation

Biofilms are complex extracellular matrix-embedded, multi-layered microbial structures on biotic or abiotic surfaces which are formed by microbe-microbe and microbe-surface interactions [50]. *C. glabrata* biofilms display antifungal resistance, and are characterized by a compact dense structure of yeast cells nested in an extracellular matrix which is composed of high levels of proteins and carbohydrates including β-1,3 glucan [50,51,52]. Large scale and candidate gene-based studies have identified several genes that are pivotal to biofilm formation in *C. glabrata* [36,37,50,53,54]. Among these, the adhesin encoded by the *EPA6* gene, which is regulated by multiple factors including the CgYak1p kinase, subtelomeric silencing, chromatin remodeling Swi/Snf complex components and the transcriptional factor CgCst6, plays a central role [36,37,53]. Additionally, other adhesins, cell wall proteins and RNA polymerase II mediator complex subunits including Epa3, Epa7, Epa12, Awp4–6, Pwp1, Pwp3, Med12, Med13 and Med15 have also been implicated in biofilm formation [36,37,40,53,54]. Gene expression profiling analysis has revealed differential expression of adhesin-encoding genes in biofilms formed under in vitro and in vivo conditions [55]. Furthermore, increased and decreased levels of stress proteins and glycolytic enzymes, respectively, have been reported during the biofilm mode of growth [56]. The ability of *C. glabrata* to form biofilms has also been found to be enhanced in the high-iron environment [57]. Moreover, recent studies have shown a close association between the capacity to form biofilms and colonize murine organs in the systemic candidiasis model [57,58], however, the precise mechanisms underlying this relationship are unknown. 

### 3.3. Aspartyl Proteases

Secreted aspartyl proteases are key fungal virulence factors [28]. However, despite the lack of any secreted proteolytic activity [59], the *C. glabrata* genome contains eleven genes (*CgYPS1-11*) that code for a family of eleven putative GPI-anchored aspartyl proteases, known as Yapsins [60]. *CgYPS* genes have been shown to be regulated by various environmental conditions including low pH, thermal stress, macrophage and neutrophil internalization [60,61,62,63]. Consistently, CgYapsins are pivotal to the regulation of several cellular processes such as maintenance of the cell wall architecture, pH and vacuole homeostasis, biofilm formation and interaction with the host [58,60,61,64]. The latter includes suppression of the production of pro-inflammatory cytokine IL-1β in human THP-1 macrophages, facilitating survival in macrophages and virulence in the models of murine systemic candidiasis and *Drosophila melanogaster* [58,60,65,66]. 

**Figure 1:**
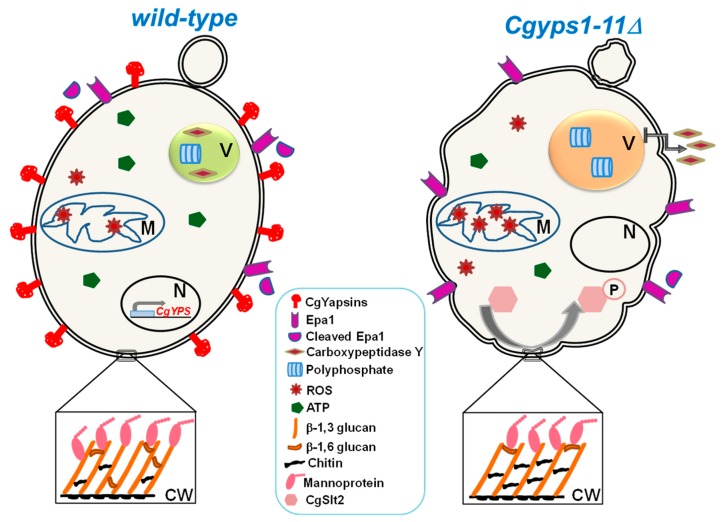
Schematic representation of pleiotropic defects associated with lack of CgYapsins. The Cgyps1-11Δ mutant displays increased ROS and diminished ATP levels, enlarged acidic vacuole with higher amount of polyphosphate, missorting of the vacuolar carboxypeptidase Y to the medium and constitutive activation of the protein kinase C-mediated cell wall integrity (CWI) pathway. CgSlt2 is the terminal MAPK of the CWI pathway. Furthermore, the cell wall of the Cgyps1-11Δ mutant contains lower amounts of β-glucan and mannan, and higher amount of chitin. Processing of the adhesin Epa1 from the cell wall is also reduced in the Cgyps1-11Δ mutant. The altered cell wall composition in the mutant may contribute to diminished biofilm formation, increased activation of macrophages and reduced colonization and virulence in the systemic candidiasis model. In line with mutant phenotypes, CgYPS genes are induced in response to diverse stresses including macrophage internalization and thermal, pH and cell wall stress. V, M, N and CW indicate vacuole, mitochondria, nucleus and cell wall, respectively.

Of eleven CgYapsins, CgYps1 is uniquely required to survive acid stress, as *CgYPS1* deletion led to low intracellular pH, high ROS (reactive oxygen species) levels and cell death under low pH and acidic environmental conditions [61]. This specific role of *CgYPS1* in intracellular pH homeostasis has been attributed, in part, to regulation of the ATPase activity of the plasma membrane proton pump, CgPma1 [61]. Additionally, the mutant lacking eleven yapsins was found to display large acidic vacuole, elevated metal ion susceptibility, mis-sorting of the vacuole hydrolase carboxypeptidase Y (CPY), diminished vacuolar ATPase activity and perturbed polyphosphate and energy homeostasis (Figure 1) [64]. Furthermore, infection with the *Cgyps11* mutant, generated by the CRISPR-Cas system, resulted in slower death of fruit flies lacking adapter of the Toll signaling pathway MyD88 [67]. Lastly, the *Cgyps1-11Δ* mutant displayed sunken cell walls containing higher chitin and reduced β-glucan and mannan levels (Figure 1) [58,64]. CgYapsins have also been implicated in shedding Epa1 adhesin off the cell wall, as Epa1 release into the medium was drastically reduced in the *Cgyps1-11Δ* mutant compared to *wild-type* cells (Figure 1) [60]. Although CgYapsins rank amongst the major virulence factors of *C. glabrata*, a direct link between the yapsin enzyme activity and different cellular processes is yet to be established.

### 3.4. Colony and Mating-Type Switching

Phenotypic plasticity is the ability of a given genotype to produce different phenotypes in varied environmental conditions [10]. *C. glabrata* is known to exhibit four different-colored colony types, white, light brown, dark brown and very dark brown, in the presence of copper sulfate or phloxine B [68]. Switching between these phenotypes, that was found to be common, spontaneous and reversible [68], had also been shown to occur at sites of colonization in vaginitis patients [69]. Moreover, *C. glabrata* is also known to undergo spontaneous reversible switching from a regular to an irregular wrinkled colony type [68]. Importantly, morphological switching from the yeast to the hyphal form has not been reported [28], although *C. glabrata* displayed pseudohyphal structures in response to nitrogen starvation and carbon dioxide exposure [70,71]. Furthermore, disruption of the transcription factor CgAce2 is known to lead to cell aggregation, due to defects in cell separation, and hypervirulence [72,73].

**Figure 2:**
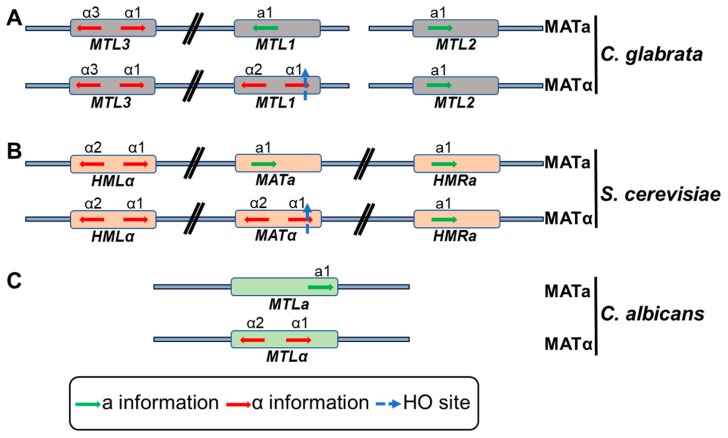
Schematic representation of the mating-type (*MAT*)-like loci in *C. glabrata* (**A**), *S. cerevisiae* (**B**) and *C. albicans* (**C**). *C. glabrata* and *S. cerevisiae* has three mating-type loci while *C. albicans* has one mating-type locus. *MTL1*, *MAT* and *MTL* loci determine the strain mating type in *C. glabrata*, *S. cerevisiae* and *C. albicans*, respectively. The other two mating-type loci in *C. glabrata* (*MTL2* and *MTL3*) and *S. cerevisiae* (*HMR* and *HML*), encoding a and α information, remain silent. *S. cerevisiae* has all three mating-type loci on the chromosome III, while the *C. albicans MTL* locus is present on the chromosome 5. In contrast, *C. glabrata* mating-type-like loci exist on two different chromosomes with *CgMTL1* and *CgMTL3*, and *CgMTL2* being present on the chromosome B and E, respectively. *S. cerevisiae* and *C. glabrata* have HO endonuclease site within the *α1* gene while *C. albicans* lack HO site.

*C. glabrata* has three mating type (*MAT*)-like loci, *CgMTL1* (*MAT*), Cg*MTL2* (*HMR*) and *CgMTL3* (*HML*), whose genomic configuration is similar to that of the *S. cerevisae MTL* loci [74]. Contrarily, *C. albicans* contains a single *MTL* locus that regulates mating and cell type (Figure 2) [75]. The *MAT* locus in *S. cerevisiae*, consisting of the active *MAT* locus, and silent HMLα and *HMRa* loci, resides on the chromosome III (Figure 2), and the mating type is determined by the presence of *MATa* or *MATα* allele [75]. However, unlike *S. cerevisiae*, the *MAT* loci are present on different chromosomes in *C. glabrata* with *CgMTL1* and *CgMTL3* on chromosome B, and Cg*MTL2* on chromosome E (Figure 2) [76]. *CgMTL1* locus encodes either ‘a’ (*Cga1*gene) or ‘alpha’ (*Cgα1* or *Cgα2* genes) information while *CgMTL2* and *CgMTL3* code for ‘a’ and ‘α’ information, respectively [74,76]. While *CgMTL1* and *CgMTL2* are transcriptionally active, *CgMTL3* is subjected to subtelomeric silencing [77]. Despite having two mating types, a and α, *C. glabrata* lacks spontaneous mating-type switching. However, ectopic expression of the *S. cerevisiae* endonuclease-encoding HO gene is known to result in efficient mating-type switching and lethality in *C. glabrata* [78]. Mating-type switching has also been reported at sites of colonization in vaginitis patients [69]. Of note, genomic recombination between different clades and evidence for an active sexual cycle have recently been reported in *C. glabrata* [25]. Altogether, the above-mentioned switching mechanisms may generate phenotypic diversity that is likely to assist *C. glabrata* in adapting to different environmental conditions.

### 3.5. Stress Response Mechanisms

*C. glabrata* is highly tolerant to oxidative, cell wall, osmotic and endoplasmic reticulum (ER) stress [79]. The environmental stress response in *C. glabrata* is governed by two transcriptional regulators CgMsn2 and CgMsn4 [38], while the oxidative stress response is regulated by the sole catalase CgCta1 [80], two superoxide dismutases CgSod1 and CgSod2 [81], and glutathione biosynthetic enzymes CgGsh1 and CgGsh2 [82,83]. *CgCTA1* expression is regulated by several stress response transcriptional regulators including CgYap1, CgMsn2, CgMsn4 and CgSkn7 [80,84,85,86]. Additionally, lack of the histone deacetylase CgHst1 led to high expression of *CgCTA1* and elevated resistance to oxidative stress, which was dependent upon CgMsn4 [87]. Despite an essential role of CgCta1 in resistance to the hydrogen peroxide-generated oxidative stress in vitro, the *Cgcta1Δ* mutant was not attenuated for virulence in the murine disseminated candidiasis model which suggests the existence of a CgCta1-independent resistance mechanism in vivo [80]. Recently, Kounatidis et al. have identified the transcriptional adapter of the Spt-Ada-Gcn5 acetyltransferase (SAGA) complex, CgAda2, by screening a library of 196 transcription factor mutants, to be essential for ROS survival in the *Drosophila* larvae model [88]. Furthermore, tryptophan-based pigment production is also known to aid in survival of the ROS stress [89].

The protein kinase C (PKC)-mediated signaling pathway safeguards the cell wall in *C. glabrata*, and its terminal mitogen-activated protein kinase (MAPK) CgSlt2, along with other cascade components, is required for survival of the cell wall stress [54,90,91]. Intriguingly, the PKC pathway is also regulated by the ER stress [92]. In addition, components of the calcineurin signaling have also been found to be essential for maintenance of the cell wall integrity [93,94]. Calcineurin signaling is also known to play a pivotal role in the virulence of *C. glabrata* [93,94]. The response of *C. glabrata* to osmotic stress is yet to be characterized in detail. However, the high osmolarity glycerol pathway [HOG] is known to be activated by sorbic acid, and its terminal MAPK CgHog1 is required for survival of weak acid and osmotic stress [95,96]. For survival of the ER stress, essentiality of the key ER stress sensor, CgIre1 endoribonuclease, was attributed to its role in activation of the non-canonical unfolded protein response pathway [97]. Furthermore, lack of CgIre1 led to diminished virulence in both immunosuppressed and immunocompetent mice [97]. Contrarily, deletion of the transcriptional co-activator CgAda2 conferred resistance to the ER stressor, tunicamycin, and hypervirulence in the immunocompromised mice [98]. Besides Ire1 signaling, two other stress-responsive pathways, PKC-mediated cell wall integrity and calcineurin signaling, have also been implicated in regulating the transcriptional response to the ER stress [94,97]. Both these signaling cascades are also required to survive the azole and echinocandin antifungal stress [54,91,99,100].

Of three common antifungal drugs, polyenes, azoles and echinocandins, for treatment of systemic *Candida* infections, *C. glabrata* is intrinsically less susceptible to azoles which impede ergosterol biosynthesis by inhibiting the cytochrome P450-dependent lanosterol 14α-demethylase enzyme [101]. Recently, a substantial number of azole-resistant *C. glabrata* isolates have also been found to be resistant to cell-wall targeting drugs echinocandins, which inhibit the β-glucan synthase enzyme [101,102,103]. Moreover, due to renal toxicity, the use of polyene antifungals, which bind to ergosterol in the plasma membrane and disrupt cell membrane functions, is largely limited [101]. Hence, several studies have been conducted to advance our understanding of the signaling pathways that *C. glabrata* utilizes to cope/counteract antifungal stress. The PKC-mediated cell wall integrity pathway has been shown to be required for the transcriptional activation of multidrug efflux pumps, which is the most frequent azole resistance mechanism in clinical settings world-wide [91,101,104,105]. Other azole resistance mechanisms include mitochondrial dysfunction and overexpression of the sterol biosynthetic target enzyme [28,101]. Resistance towards echinocandins is primarily due to mutations in the *CgFKS1* and *CgFKS2* genes that code for β-1,3-glucan synthase [103]. Both calcineurin and PKC signaling have been implicated in echinocandin resistance in *C. glabrata* [93,103,106]. Polyene resistance is less frequent in *C. glabrata*, and mutations in genes encoding an ergosterol biosynthetic enzyme Erg6 and components of the mismatch repair pathway have been associated with polyene resistance [103,107,108,109]. Because of space limitations, the factors, which confer tolerance and/or resistance to antifungal drugs in *C. glabrata* are not discussed here; readers may refer to recent reviews on this topic [101,103].

Overall, the high resistance to diverse stressful conditions may significantly contribute to the survival of *C. glabrata* in varied host niches.

### 3.6. Nutrient Acquisition Pathways

A prerequisite to thrive in the host is the ability to acquire nutrition from the nutrient-limited host environment. *C. glabrata* has rewired its metabolic machinery, and developed novel nutrient uptake mechanisms. It has lost 5, 4, 3, 3 and 6 genes involved in galactose metabolism, phosphate metabolism, cell rescue, defense and virulence, nitrogen and sulfur metabolism, and allantoin catabolism, respectively [17]. *C. glabrata* also lacks genes involved in thiamine, pyridoxine and nicotinic acid biosynthetic pathways [28]. The loss of nicotinic acid synthesis genes was thought to be due to close association of *C. glabrata* with the mammalian host, however, recent studies report that the nicotinic acid auxotrophy is a trait of the *Nakaseomyces* clade, which contains both environmental and pathogenic species [18].

#### 3.6.1. Carbon Acquisition

*C. glabrata* is a facultatively anaerobic, crabtree-positive yeast which prefers fermentation over respiration in the presence of oxygen [110,111]. *C. glabrata* can assimilate both glucose and trehalose, but lacks genes for assimilation of galactose and sucrose [17,28]. Importantly, *C. glabrata* can utilize l-amino acids such as glutamate, aspartate and proline as the sole carbon and nitrogen source [112]. *C. glabrata* is also able to grow in very low concentration of glucose, and its glucose sensing pathway resembles to that of *S. cerevisiae* [113]. The *C. glabrata* genome encodes eleven hexose transporters, two transcriptional regulators CgRgt1 and CgMig1, and two glucose sensors, CgRgt2 and CgSnf3. CgSnf3 acts as the high-affinity glucose sensor which is essential for growth under glucose-limited conditions and in macrophages [113]. *C. glabrata* also has a set of six conserved duplicated gene paralogs encoding glycolytic enzymes, Eno1/Eno2, Pyc1/Pyc2, Glk1/Emi2, Hxk1/Hxk2, Tdh2/Tdh3 and Cdc19/Pyk2 [18], that may aid in the increased carbon flux through glycolysis, and contribute to its respiro-fermentative lifestyle [114].

#### 3.6.2. Phosphate Acquisition

The macronutrient phosphorus, in the form of inorganic phosphate, is pivotal to the biosynthesis of cellular moieties and regulation of various metabolic processes. The PHO (phosphate signal transduction) pathway executes the phosphate starvation response in *C. glabrata* via the transcription factor CgPho4, which itself is regulated by the cyclin (CgPho80)/cyclin-dependent protein kinase (CDK; CgPho85)/CDK inhibitor (CgPho81) complex [115]. However, the *C. glabrata* genome lacks an ortholog of the *S. cerevisiae PHO5* gene which codes for a phosphate starvation-inducible acid phosphatase [115]. Instead, *C. glabrata* genome uniquely codes for a family of three phosphatases, CgPmu1-3 (*C. glabrata* phosphomutase-like protein 1–3), which possess different substrate specificity [116]. CgPho4 was found to be required for the phosphate starvation-induced phosphatase activity, and deletion of the putative CgPho4 nuclear exporter, CgMsn5, led to increased phosphatase activity under phosphate-surplus conditions [115]. Of three CgPmu proteins, secreted phosphatase activity of only CgPmu2 was induced in response to phosphate starvation [116]. Consistent with this, *CgPMU2* transcript levels were lower in high-phosphate conditions and substantially higher during phosphate starvation [116]. Furthermore, *CgPMU2* and *CgPHO* gene expression was found to be regulated by CgPho4 in a largely CgPho2 coactivator-independent manner [115,117]. This reduced dependence on CgPho2 appears to have substantially broadened the target gene set of CgPho4, which included genes involved in phosphate homeostasis as well as adherence, cell wall biosynthesis, non-phosphate-related stress response and carbohydrate metabolism [118]. Recently, CgPmu3 has been shown to be a thiamine phosphatase that is transcriptionally regulated by the transcription factor CgThi3 in response to thiamine starvation [119]. Overall, *C. glabrata* appears to have neofunctionalized the CgPmu family to compensate for the loss of the ancestral Pho5 phosphatase [116,119]. Although CgPmu2 was required to grow in the presence of organic phosphate compounds as the sole phosphate source in vitro [116], it remains to be determined whether neofunctionalization of the *CgPMU* gene family confers a growth advantage in the mammalian host.

#### 3.6.3. Iron Acquisition

Of known fungal iron uptake mechanisms, siderophore-mediated uptake of Fe^3+^, reductive iron acquisition, haemoglobin/haem uptake [120], all systems are operational in *C. glabrata* except for the receptor-mediated haem uptake [121,122]. The sole xenosiderophore transporter CgSit1 typifies the siderophore-mediated iron uptake system in *C. glabrata* [123]. CgSit1 has been shown to be pivotal to survival in the iron-limited host environment [123]. Although *C. glabrata* exhibits haemolytic activity in vitro and possesses hemolysins, it lacks the haem receptor [121,122,124]. *C. glabrata* is also unable to utilize the host iron proteins, haemoglobin, and transferrin, as iron sources [125]. Recently, putative cell surface-associated, cysteine-rich Common in Fungal Extracellular Membrane (CFEM) domain-containing protein (CgCcw14), haem oxygenase (CgHmx1) and intracellular iron trafficking machinery components including vacuolar (CgCcc1, CgSmf1, CgSmf3, and CgFth1) and mitochondrial (CgMmt1, CgMmt2, CgAtm1, CgMrs3, and CgMrs4) iron transporters have been identified in *C. glabrata* [122,125], however, their role in iron metabolism is yet to be fully characterized.

The reductive iron acquisition system in *C. glabrata* consists of three ferric reductases, multicopper ferroxidase CgFet3 (oxidizes Fe^2+^ to Fe^3+^), an iron transporter/permease CgFtr1 (facilitates the passage of Fe^3+^ across the membrane) and a copper ion transporter CgCcc2 (loads copper on to CgFet3) [122]. Disruption of the high-affinity iron uptake components resulted in perturbed iron homoeostasis, debilitated survival under in vitro iron-limiting conditions and attenuated virulence [122]. Despite the presence of three ferric reductase genes, *C. glabrata* exhibited no surface ferric reductase activity, and it has been postulated that extracellular ferric reduction may be achieved through a secreted molecule [125]. *C. glabrata* could also utilize ferritin and ferric chloride as iron sources in a pH-dependent manner via reductive high-affinity iron uptake system [125].

Furthermore, *C. glabrata* has been reported to respond to iron-deplete condition via expression of the high-affinity iron permease CgFtr1 on the plasma membrane, and to iron-replete condition by trafficking CgFtr1 to the vacuole [57]. This retrograde trafficking of CgFtr1 from the plasma membrane to the vacuole is dependent on the sole class III phosphoinositide 3-kinase kinase, CgVps34 [57]. With regard to signaling pathways, Hog1-mediated MAPK has been shown to be essential for survival under high iron stress, as a lack of CgHog1 resulted in elevated intracellular iron and mitochondrial iron content and cell death in the high-iron environment [96].

**Figure 3:**
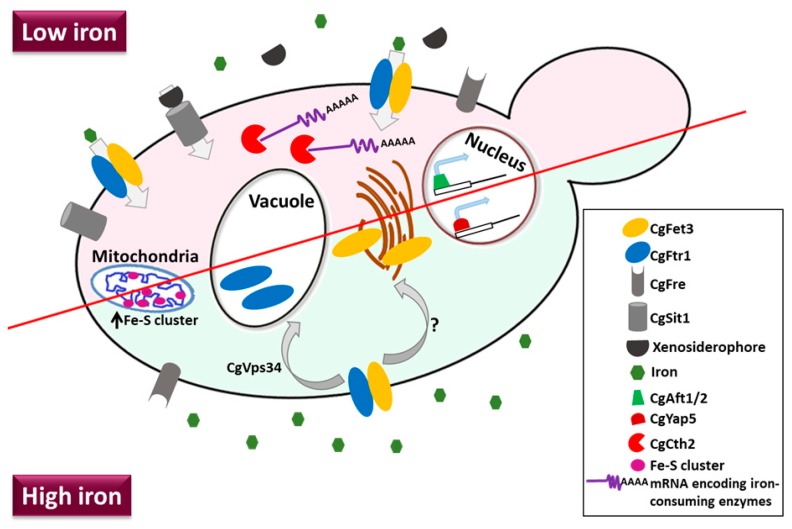
A schematic depicting major iron acquisition and homeostasis pathways in *C. glabrata*. The iron acquisition mechanisms of *C. glabrata* include the high-affinity iron uptake system mainly comprised of the CgFtr1 permease and the CgFet3 ferroxidase, and the siderophore uptake system consisting of the sole xenosiderophore transporter CgSit1. In response to low-iron, genes of the reductive iron acquisition system are upregulated in the transcriptional factor CgAft1/2-dependent manner. Additionally, the CgCth2 protein, in an iron-limited environment, degrades mRNAs that code for iron-consuming enzymes. Contrarily, *C. glabrata* responds to high iron by retrograde trafficking of the components of the high-affinity iron uptake system from the plasma membrane. CgFtr1 is transported to the vacuole in a phosphoinositide-3-kinase (CgVps34)-dependent manner, while CgFet3 is trafficked to an intracellular organelle. The factor/s responsible for the retrograde trafficking of CgFet3 are unknown. In addition, the transcriptional factor CgYap5 regulates genes involved in the iron-surplus response, and iron-sulfur cluster biogenesis is upregulated in the high-iron environment. The functions of ferric reductases (CgFre) are yet to be deciphered, as *C. glabrata* does not exhibit cell surface-associated ferric reductase activity.

The iron regulon in *C. glabrata* is comprised of a set of 51 genes that undergo reciprocal regulation in response to low and high environmental iron conditions [96]. *C. glabrata* possesses a unique hybrid iron regulatory network that consists of orthologs of the *S. cerevisiae* positive master iron regulator Aft1 and mRNA-encoding iron-requiring enzyme-degrading protein Cth2, as well as the *C. albicans* positive iron regulator Sef1 [126]. Of note, three bZip transcription factors, CgYap1, CgYap5 and CgYap7 have also been implicated in regulation of the heme biosynthesis, iron-excess stress response and iron-sulfur cluster biogenesis, respectively [127]. Additionally, CgHap5, a subunit of the CCAAT-binding complex, was found to interact with CgYap5, as well as being pivotal to the CgYap5-mediated iron stress response [128]. Figure 3 depicts major iron acquisition pathways in *C. glabrata*.

## 4. *C. glabrata*-Host Interaction

During its commensal and pathogenic life style, *C. glabrata* is thought to interact with host epithelial, endothelial and immune cells [10]. The in vivo and in vitro models, that have largely been used to study the pathogenesis of *C. glabrata*, include *Mus musculus* (mice), *D. melanogaster* (fruit fly) and *Galleria mellonella* (wax moth), and epithelial and endothelial cell lines, macrophage cell lines, human neutrophils and reconstituted oral epithelia, respectively [23,60,89,129,130,131,132]. A brief account of *C. glabrata*-host cell interaction is described below.

### 4.1. C. glabrata-Epithelial Cell Interaction

Adhesion of *C. glabrata* to epithelial cells and extracellular matrix is a prerequisite for mucosal colonization. Although the in vitro adherence of *C. glabrata* to epithelial cells is primarily mediated by the Epa1 adhesin [32], Epa6 and Epa7 have been shown to be pivotal to adherence under specific environmental conditions [30,36]. Epa6 is also known to mediate binding of *C. glabrata* to the human extracellular matrix protein, fibronectin [133]. Furthermore, increased adherence to Chinese Hamster Ovary derived-Lec2 cells has been reported for *C. glabrata* strains carrying the hyperactive *CgPDR1* allele, which was attributed to the elevated expression of Epa1 [34]. Unlike *C. albicans*, *C. glabrata* elicited production of the granulocyte monocyte colony-stimulating factor (GM-CSF) in oral epithelial cells but caused no significant cytotoxicity [129]. This GM-CSF production in oral epithelial cells was later shown to be dependent upon the lactosylceramide receptor CDw17-mediated activation of NF (nuclear factor)-kB [134]. Similarly, *C. glabrata* could neither degrade the E-Cadherin protein present in the adherens junctions of the oral mucosal epithelium [135] nor invade the reconstituted human oral epithelium in vitro [130]. However, *C. glabrata* cells producing tryptophan-based pigment have been reported to cause increased damage to human oral epithelial TR146 cells [89]. A recent study has shown *C. glabrata* to induce phosphorylation of the ephrin type-A receptor 2 (EphA2), that binds to β-glucan, in immortalized normal human oral keratinocytes (OKF6/TERT-2) [136]. Furthermore, although the Toll-like receptor 2 (TLR-2) has also been implicated in recognition of *C. glabrata* and induction of the NF-κB-dependent release of TNF-α and IL-6 cytokines in rat tracheal epithelial cells [132], more studies are required to better understand the immune response of epithelial cells to association with *C. glabrata*.

### 4.2. C. glabrata-Endothelial Cell Interaction

The two main events that lead to systemic infection are dissemination (entry of the pathogen into the bloodstream) and tissue invasion (entry into surrounding tissues from the bloodstream). In an in vitro model of the human umbilical vein endothelial cells (HUVEC), *C. glabrata* was found to be able to cross the endothelial barrier [137]. Furthermore, two GPI-anchored cell wall proteins, CgPwp7 and CgAed1, have been shown to be required for adherence to HUVEC, as mutants lacking these adhesins were 2-3-fold less adherent [23]. However, unlike *EPA* genes, *CgPWP7* and *CgAED1* were not transcriptionally regulated by silencing [23]. In addition, two enzymes of the N-linked glycosylation system, α-1,6-mannosyltransferase (CgAnp1) and α-1,2-mannosyltransferase (CgMnn2), have also been implicated in adherence, as mutants lacking these enzymes were hyperadherent to the human microvascular endothelial cells HMEC-1 [138]. The *C. glabrata Cgsir3Δ* mutant, which expresses higher levels of Epa adhesins [36], has been reported to display mannose- and galactose-dependent strong binding to the coronary endothelium, through the coronary endothelial luminal membrane lectinic G protein-coupled receptors (GPCRs) including endothelin-2 and α-adrenergic 1A receptor, that led to altered cardiac functions [139]. Although this study underscored the binding of GPCRs to *C. glabrata*, *C. glabrata*-endothelial cell interaction are yet to be analyzed in depth.

### 4.3. C. glabrata-Neutrophil Interaction

Neutrophils are major players of the host defense system against fungal infections [140]. However, the role of neutrophils in the control of *C. glabrata* infections is not well-studied. Human neutrophils have been reported to release neutrophil extracellular traps after engulfment of *C. glabrata* cells [141]. Additionally, human neutrophils, after phagocytosis, killed and dumped *C. glabrata*, which may aid in activation of the immune response [142]. Intriguingly, *C. glabrata* was preferentially taken up by monocytes in the whole blood infection model, and infiltration of predominantly monocytes was observed in mouse kidneys [143]. Consistent with this, *C. glabrata*-activated neutrophils secreted monocyte chemoattractants, MIP-1α and MIP-1β, leading to increased migration of monocytes to the site of neutrophil-*C. glabrata* confrontation [143]. However, neutrophils of the dectin-2^-/-^ knock out mice, which displayed elevated susceptibility to *C. glabrata* infections, have been reported to mount a deficient oxidative burst, pointing towards a role of the dectin-2 receptor and neutrophils in the control of *C. glabrata* infections [144]. The response of *C. glabrata* to the neutrophil environment has also been studied with genes involved in oxidative stress, gluconeogenesis, glyoxylate cycle, and methionine metabolism displaying upregulation [63]. Although a pigment derived from tryptophan is known to protect *C. glabrata* against the neutrophil attack [89], a detailed characterization of *C. glabrata*-neutrophil interaction is yet to be done.

### 4.4. C. glabrata-Natural Killer Cell Interaction

A recent study has highlighted the role of Natural Killer cells, which are effector lymphatic cells of the innate and adaptive immune system, in combating *C. glabrata* infections [145]. The Epa1, Epa6 and Epa7 adhesins were shown to be specifically recognized by the NKp46/NCR1 receptor, resulting in the clearance of disseminated infection [145]. Additionally, the murine dendritic cells have also been reported to produce IFN-β through the toll-like receptor TLR7, thereby, underscoring the role of IFN-I signaling in modulation of the host response to *C. glabrata* infection [146].

### 4.5. C. glabrata-Macrophage Interaction

Macrophages are the primary effectors of the innate immune system [140]. Besides engulfing and killing the fungal pathogen, they also facilitate recruitment of other immune cells, through cytokine and chemokine production, at the site of infection [140]. *C. glabrata* survives and replicates in human and murine macrophages without adversely affecting macrophages [60,65,147]. Among the pathogen recognition receptors, the C-type lectin receptors, dectin 1 and dectin-2, which recognize cell wall β-glucan, and α-mannan and β-glucan, respectively, have been implicated in the recognition of *C. glabrata* [144,148]. *C. glabrata* infection did not substantially activate any MAPK pathway including Erk1/2 (Extracellular signal-regulated kinases), SAPK/JNK (Stress-activated protein kinases/Jun amino-terminal kinases) and NF-κB signaling [149]. In accordance, macrophages did not produce TNF-α, IL-6, IL-8, IL-12, and IFN-γ pro-inflammatory cytokines, however, GM-CSF production has been reported upon *C. glabrata* infection [65]. Additionally, the spleen tyrosine kinase, Syk, is known to be phosphorylated in response to *C. glabrata* infection which resulted in the NLRP3 inflammasome-dependent production of IL-1β in human THP-1 macrophages [58]. The family of eleven cell surface-associated proteases (CgYapsins) has been shown to be required to keep the Syk pathway activation in check, as lack of these proteases led to increased IL-1β release and killing of *C. glabrata* cells [58].

**Figure 4:**
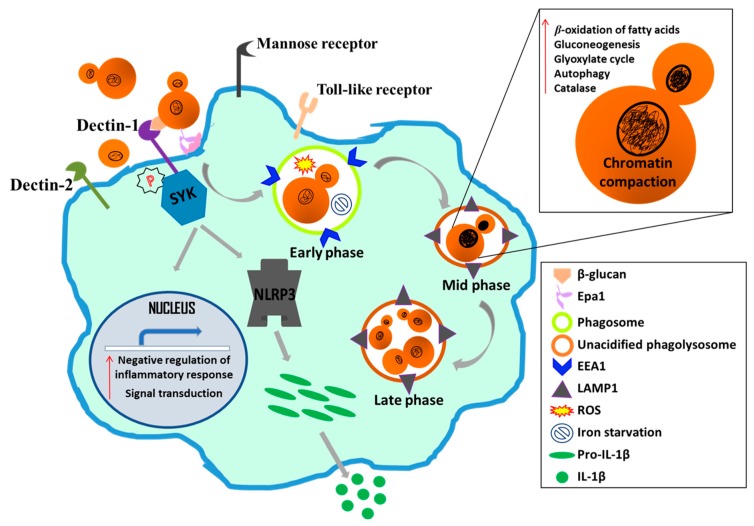
A schematic illustrating major facets of *C. glabrata*-macrophage interaction. *C. glabrata* cells are internalized by macrophages probably via the Dectin receptor-mediated endocytosis. The *C. glabrata* Epa1 adhesin also facilitates binding to macrophages. After phagocytosis, *C. glabrata* first resides in the early endosome [decorated with EEA1 (Early Endosome Antigen 1)] and later in the late endosome [marked with LAMP1 (Lysosomal-associated membrane protein 1)], with cell replication occurring in the unacidified phagolysosome. During the course of intracellular proliferation, *C. glabrata* remodels its chromatin, activates autophagy and upregulates genes involved in the β-oxidation of fatty acids, gluconeogenesis, glyoxylate cycle and oxidative stress. Metabolic and stress pathways reconfiguration helps *C. glabrata* survive the macrophage internal milieu which is limited for iron and glucose, and contains high levels of ROS. On the other hand, the infected macrophage weakly activates the Dectin-mediated Syk signaling through Syk phosphorylation, and secretes small amount of the pro-inflammatory cytokine IL-1β in a Syk-NLRP3 inflammasome-dependent manner. The transcriptional response of the macrophage to *C. glabrata* internalization primarily consists of induction of genes involved in signal transduction and negative regulation of the inflammatory response and cytokine secretion processes.

*C. glabrata* is also known to impede maturation of the phagosome in macrophages [65,147], and the phosphoinositide 3-kinase subunits (CgVps15 and CgVsps34), mannosyltransferases (CgMnn10 and CgMnn11) and vesicular trafficking proteins (CgLdb17 and CgSla2) contributed to the inhibition of phagolysosomal acidification [147,149,150]. Of note, CgMnn10 and CgMnn11 have also been implicated in the ammonia extrusion-dependent environmental alkalinization which may partly account for their role in impeding phagolysosomal acidification [149]. *C. glabrata* activates pexophagy and autophagy processes, undergoes chromatin remodeling and evades immune responses to survive and replicate in macrophages [84,147,150]. Lastly, despite encountering iron restriction, ROS and carbon starvation, *C. glabrata* has equipped itself well with strategies to proliferate in macrophages, including transcriptional reconfiguration of cellular pathways [60,65,147]. Genes belonging to the glyoxylate cycle, β-oxidation of fatty acids, gluconeogenesis, methyl citrate cycle and proteolysis have been shown to be upregulated, while genes encoding glycolytic enzymes and ribosomal translational machinery components were found to be downregulated in macrophage-internalized *C. glabrata* cells [60]. Furthermore, the continuous contact with macrophages during microevolution studies led to a change in the morphology of *C. glabrata* cells from yeast to pseudohyphae which was attributed to a mutation in the chitin synthase-encoding gene *CgCHS2* [151]. Altogether, because of its ability to subvert the immune response and replicate in macrophages in vitro, macrophages are assumed to be the Trojan horses for *C. glabrata* [58,143,151]. Figure 4 summarizes key aspects of *C. glabrata*-macrophage interaction.

## 5. Conclusions

*C. glabrata* occupies a unique position in the phylogenetic tree and appears to possess requisite attributes to establish successful infections in the human host. However, it is significantly less pathogenic than *C. albicans*. The lack of invasive hyphal forms, secreted proteolytic activity and invasins, and limited nutrient plasticity including non-utilization of haemoglobin as an iron source are likely to contribute to the low pathogenicity of *C. glabrata*. Research over the last two decades has brought many unexpected biological features of *C. glabrata* to the fore. Owing to its reduced susceptibility to azole antifungals and emerging resistance to echinocandins, effective treatment of *C. glabrata* infections remains a clinical challenge. Hence, future research, focusing on adhesins, proteases, stress response regulators and nutrient acquisition machinery, that may modulate interaction with the host, is likely to elucidate precise mechanisms underneath the commensal and the opportunistic life style of this important pathogen.

## Figures and Tables

**Table 1:** Comparison of morphological and pathogenesis traits of *C. glabrata* and *C. albicans*.

Feature	*Candida glabrata*	*Candida albicans*
Ploidy	Haploid	Diploid
Cellular morphology	Yeast	Yeast, pseudohyphae and hyphae
Cell size	1–4 µm	4–6 µm
Phylogeny	Non-CTG clade	CTG clade
Phenotypic switching	Present	Present
Carbon assimilation	Glucose and trehalose	Glucose, trehalose, maltose and galactose
Auxotrophy	Niacin, thiamine, pyridoxine	None
Crab tree	Positive	Negative
Mitochondrial function	Petite positive	Petite negative
Mating genes	Present	Present
Haem receptor	Absent	Present
Haemoglobin and transferrin utilization	Absent	Present
Innate azole resistance	Present	Absent
Secretory aspartyl proteases	Absent	Present
Life style	Probably commensal, and pathogenic	Commensal and pathogenic
Major sites of infection	Vaginal, oral, disseminated	Vaginal, oral, disseminated
Major adhesins	Lectins (Epa)	Lectins (Als and Hwp)
Biofilm	Present	Present
Invasion	Not known	Induced endocytosis and active penetration
Damage to host cells	No significant damage	Substantial damage
